# Analysis of responder-based endpoints: improving power through utilising continuous components

**DOI:** 10.1186/s13063-020-04353-8

**Published:** 2020-05-25

**Authors:** James Wason, Martina McMenamin, Susanna Dodd

**Affiliations:** 1 Population Health Sciences Institute, Newcastle University, Baddiley-Clark Building, Newcastle upon Tyne, NE2 4BN UK; 2 MRC Biostatistics Unit, University of Cambridge, Institute of Public Health, Robinson Way, Cambridge, CB2 0SR UK; 3 Department of Biostatistics, University of Liverpool (a member of Liverpool Health Partners), 1-5 Brownlow Street, Liverpool, L69 3GL UK

**Keywords:** Augmented binary method, Composite endpoint, Efficiency, Responder analysis, Statistical analysis

## Abstract

**Background:**

Clinical trials and other studies commonly assess the effectiveness of an intervention through the use of responder-based endpoints. These classify patients based on whether they meet a number of criteria which often involve continuous variables categorised as being above or below a threshold. The proportion of patients who are responders is estimated and, where relevant, compared between groups. An alternative method called the augmented binary method keeps the definition of the endpoint the same but utilises information contained within the continuous component to increase the power considerably (equivalent to increasing the sample size by > 30%). In this article we summarise the method and investigate the variety of clinical conditions that use endpoints to which it could be applied.

**Methods:**

We reviewed a database of core outcome sets (COSs) that covered physiological and mortality trial endpoints recommended for collection in clinical trials of different disorders. We identified responder-based endpoints where the augmented binary method would be useful for increasing power.

**Results:**

Out of the 287 COSs reviewed, we identified 67 new clinical areas where endpoints were used that would be more efficiently analysed using the augmented binary method. Clinical areas that had particularly high numbers were rheumatology (11 clinical disorders identified), non-solid tumour oncology (10 identified), neurology (9 identified) and cardiovascular (8 identified).

**Conclusions:**

The augmented binary method can potentially provide large benefits in a vast array of clinical areas. Further methodological development is needed to account for some types of endpoints.

## Background

In clinical trials gathering evidence about the effectiveness of a medical intervention, it is necessary to specify a primary endpoint. An endpoint should represent how patients respond after being given the treatment; it should be expected that the distribution of the endpoint will be more favourable if a treatment is effective than if it is ineffective. In many disorders it is difficult to specify just one endpoint, as an intervention may have a variety of effects that cannot be adequately measured through one measurement. For this reason, it is common in many conditions to combine multiple distinct endpoints (which we will refer to as components) into a composite endpoint.

Composite endpoints have been recommended when there is large variability in the disease manifestation, e.g. in complex multisystem diseases, allowing multiple equally relevant outcomes to be considered without the need to correct for multiplicity. They have also been advocated for rare diseases, where they might improve the power by increasing the number of events observed. On the other hand, composite endpoints have been criticised for making trial results more difficult to interpret [1].

One specific type of composite endpoint is a composite responder endpoint, which divides patients into responders and non-responders on the basis of the set of components. Some of these components may be binary (present or absent), and some may be continuous. In the case of continuous components, some dichotomisation is necessary, so that patients are responders only if the continuous component is above or below a specified threshold. In Table 1, we provide examples of some commonly used responder-based endpoints and their definitions. In some cases (such as tumour response in Table 1) a patient must meet all the criteria to be a responder; in other cases a patient must meet a set number. Some responder endpoints are not composite and are just formed from a single dichotomised continuous endpoint.

**Table 1:** Examples of responder endpoints used in different areas of medicine; italicised components denote continuous dichotomisations. To be a responder, all numbered components are required to be met

Clinical area	Endpoint	Components and definitions
Oncology	Tumour response	*1. Sum of longest diameter of target tumour lesions ≥ 30% shrinkage from baseline* 2. No new tumour lesions
Rheumatology	ACR20	*1. Swollen joint count ≥ 20% improvement* *2. Tender joint count ≥ 20% improvement* 3. 20% improvement in at least three of: *a. Patient assessment* *b. Physician assessment* *c. Pain scale* *d. Disability/functional questionnaire* *e. Acute phase reactant (ESR or CRP)* 4. No rescue therapy given
Type II diabetes	Diabetes remission	*1. Glycated haemoglobin A*_*1c*_*concentration ≤ 6.5%* *2. Fasting glucose concentration ≤ 5.6 mmol/L* 3. No non-study pharmacological treatment given

*ACR20* American College of Rheumatology 20% improvement, *ESR* erythrocyte sedimentation rate, *CRP* C-reactive protein

Responder endpoints are appealing, as they simplify several (potentially complex) pieces of information into one responder/non-responder variable. The proportion of patients who are responders serves as an easy-to-interpret measurement of the effectiveness of a treatment.

From a statistical point of view, however, this appealing simplicity comes at a non-appealing cost when one or more components are continuous. Dichotomising continuous variables loses information, a point which has been made several times (see, e.g. [2–4]). This means that when considering one continuous endpoint, it is substantially more efficient to analyse it as a continuous variable rather than dichotomise it and test it as a binary variable. As a rule of thumb, the minimum cost of dichotomisation is requiring a 35% higher sample size for the same level of statistical precision [2].

Assuming that avoiding dichotomisation is desirable, it is not obvious how this is possible when the responder endpoint consists of a mix of continuous and binary components. One method would be to use the approaches of Lachenbruch [5] or Hu and Proschan [6], which use separate test statistics for each component and form an overall test through appropriate weighting. However, this technique loses the clinical interpretability of the endpoint and does not allow efficient estimation of the probability of response. Even in the case of a single continuous component, there may be compelling clinical reasons to keep a responder endpoint dichotomised [7]: ease of interpretation to researchers and patients, wide acceptance as an important, meaningful clinical diagnosis (e.g. diabetes or hypertension).

This motivates statistical methods that can be used to keep what is clinically relevant by inferring the proportion of patients who are responders, but also utilise information contained in continuous components to improve the efficiency. For the single-component responder, this idea dates back to the 1990s, in studies where Suissa and Blais [8, 9] proposed methods for doing this for a single continuous component case. To our knowledge, this method rarely is applied in practice despite its advantages over analysing the endpoint as binary. More recently, an approach known as the *augmented binary method* has been developed that allows composite responder endpoints (that consist of at least one continuous component) to be analysed in a more efficient way, whilst maintaining the definition of the endpoint.

In this paper (and its associated supplementary material) we first describe the augmented binary method, focusing on its advantages and drawbacks. The main novel contribution of the paper is a review that identifies new clinical areas where trial efficiency can be improved through use of the augmented binary method. Finally, we discuss some further developments to the method that are motivated by the review.

## The augmented binary method: intuition, benefits and drawbacks

The augmented binary method extends previous work focused on a single dichotomised continuous endpoint [8, 9] to composite responder endpoints with a mixture of continuous and binary endpoints. The original motivation was solid tumour oncology [10, 11], but subsequent papers have focused on developing the methodology for rheumatology [12] and rare diseases using composite endpoints [13].

For simplicity we focus on the case of a composite responder endpoint that combines a dichotomised continuous component with a binary component. For example, response in solid tumour oncology consists of the sum of target lesion diameters shrinking by at least 30% from a baseline scan (dichotomised continuous) and no new tumour lesions appearing on a scan (binary). The traditional, binary analysis would work with the data on whether or not each patient is a responder or not. If a patient meets the criteria, he/she is a responder, otherwise not. If analysing a randomised controlled trial (RCT), then one might test for a difference between arms in the proportion of patients who are responders with an established method that gives an effect size, confidence interval and *p* value (e.g. logistic regression).

**Fig. 1:**
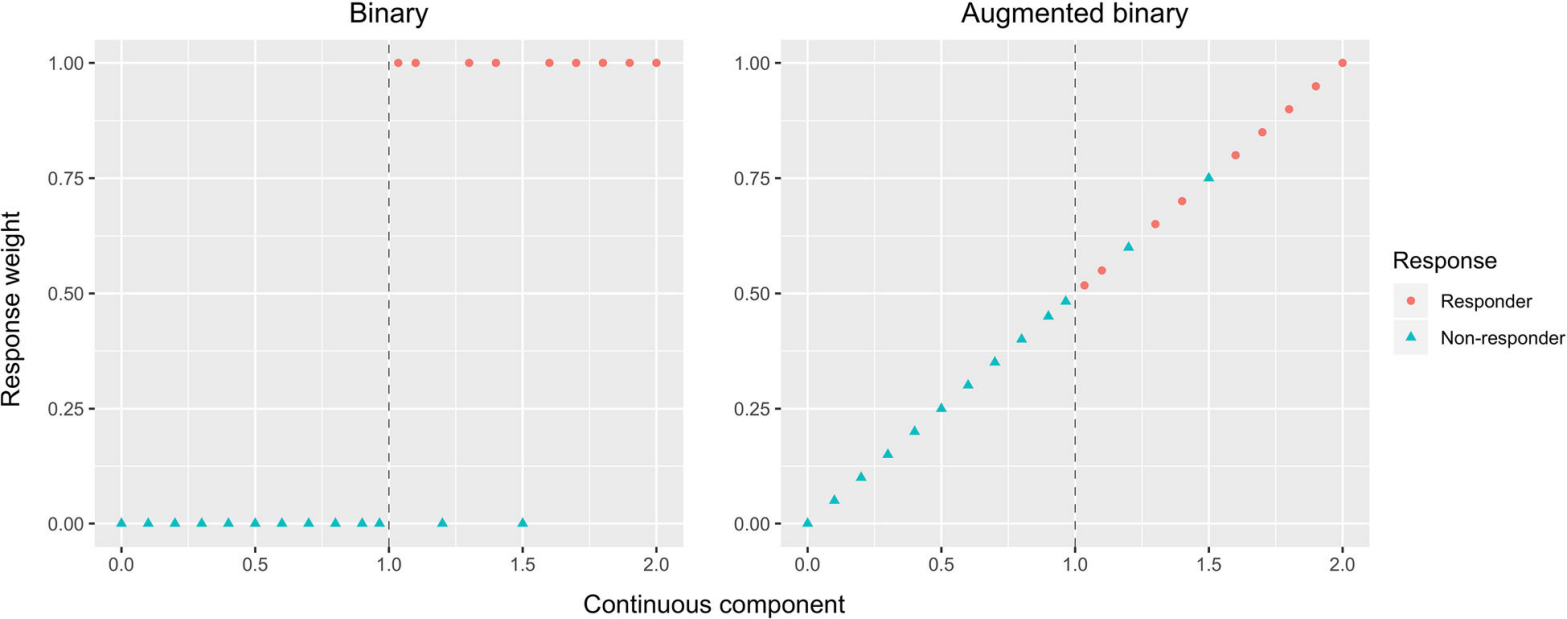
Illustration of how (hypothetical) response information from patients is weighted by the two different methods. Non-responders consist of those in whom the continuous component is below 1 and those who do not respond according to another binary criterion. Underlying the augmented binary method is a joint model that is fitted to the continuous and binary data and yields fitted ‘response weights’ for each patient; these can then be compared between arms

A detailed description of how to fit the method is provided in the supplementary material, including R code that can be used for the case of a composite responder endpoint formed from a single continuous and single binary component. The main intuition behind the method is to first fit a more sophisticated model to the data from the different components, and second to use this model to estimate a probability of response and test for a difference between arms. The second step can be thought of as weighting the different patients as a proportion of a response with this proportion depending on how close the continuous component was to the threshold. This is demonstrated in Fig. 1, which shows how patients are measured on a continuous and a binary component. The continuous measurement must be above 1 for the patient to be a responder; however, patients must also meet additional binary criteria. The binary method treats the information as 0’s and 1’s, whereas the augmented binary method uses a ‘response weight’ which is determined from the underlying model and is higher as the continuous component increases. The supplementary material contains a link to an R package that can be used to fit the model.

The benefit of the method is primarily the increased power. By better using the available information, the proportion of patients who respond (and therefore any differences between arms in an RCT) can be estimated more precisely. In more statistical language, the variance of the estimate is lower, and the width of the confidence interval (CI) is narrower. Simulation studies presented by Wason and Seaman [10] found that the average gain in power was equivalent to increasing the sample size by at least 30%. The gain can be considerably higher depending on the scenario, predominantly depending on how well the dichotomisation point divides patients. This gain in power has been confirmed by a re-analysis of the Oral Syk Inhibition in Rheumatoid Arthritis (OSKIRA-1) trial [14], which showed that the reduction in CI width was equivalent to an increase in sample size of > 50% [12]. It should be emphasised that this gain in power does not rely on additional data being collected—it just comes from using the existing data more efficiently.

There are some additional benefits of the approach. First, due to the underlying model being fitted, it better allows for missing data on different components [10] (it is generally not obvious how to handle missing data on a specific component of a composite outcome). This is especially true when there is the possibility of some components having more missing data than others. Second, it may also help address issues of misclassification due to measurement error: if a patient is truly close to the responder threshold, then a measurement error will have a potentially very large impact on the binary method, but a small impact on the augmented binary method.

There are also drawbacks. First, it is undoubtedly more complex to apply the method compared with standard binary approaches. Some code is available (see the supplementary material) for applying the method in specific cases, but a more generic implementation in different commonly used statistical programs is a high priority for the future. Second, the method makes more assumptions, for instance that the distribution of the continuous components is normal. This means that it is necessary to check this prior to analysing the data and use a suitable correction if assumptions are not met, such as applying a Box-Cox transformation [15] to ensure that the continuous component is normally distributed. Third, if the number of components or number of timepoints at which the endpoint is measured is large, applying the method can require a large amount of computational time. This is generally not an issue for an analysis of a single trial; however, assessing the performance of the method on a large number of computer simulations can become infeasible.

Up to now, the method has been applied to datasets in solid tumour oncology [10, 11], rheumatoid arthritis [12] and systemic lupus erythematosus (SLE) [16]. Based on our personal experience of peer-reviewing clinical trial papers and discussion with a wider group of clinicians, we hypothesised that there might be a much greater number of diseases where the augmented binary method could be useful. We decided that a more systematic attempt to identify these clinical areas was warranted.

## Materials and methods

We made use of the COMET (Core Outcome Measures in Effectiveness Trials) database (http://www.comet-initiative.org/resources), which lists completed and ongoing projects in core outcome set (COS) development. The COS represents the minimum that should be measured and reported in all clinical trials of a specific condition [17, 18].

We reviewed physiological and mortality trial outcomes (categorised according to [19]) recorded within all COSs in the COMET database that were published before 2016. The restriction to 2016 allowed us to utilise the COS taxonomy presented in Dodd et al. [19], which considered COSs up until then. This led to 287 COS papers to review, which were split amongst the three authors (JW, MMM and SD) to review. Each COS paper was reviewed to determine if any responder (composite or categorised continuous) endpoints were recommended for reporting in all clinical trials within that condition. In some cases, a potentially relevant endpoint was not clearly described in the core outcome paper. In this case, we examined RCTs that had used the endpoint to determine whether it was a suitable responder endpoint.

## Results

This process allowed us to identify 39 clinical areas (additional to solid tumour oncology, rheumatoid arthritis and SLE) where the augmented binary method could be utilised to gain efficiency. An additional 28 clinical areas had used responder endpoints formed from a single categorised/dichotomised continuous variable. Table 2 breaks down the number by clinical classification. A full listing of these clinical areas is given in the supplementary material. These are given by clinical classification in supplementary tables 1a–m.

**Table 2:** Number of new clinical areas identified by classification; full list provided in supplementary material

Classification	Number of conditions with suitable composite responder endpoints	Number of conditions with single-variable responder endpoints
Bleeding and transfusion	2	1
Cancer^a^	6	4
Cardiovascular and circulation	5	3
Dentistry and vision	2	1
Gastroenterology	3	1
Infectious diseases	3	0
Lungs and airways	0	2
Mental health and addiction	3	1
Neurology	2	7
Orthopaedics and trauma	1	3
Renal and urology	2	1
Rheumatology	8	3
Unclassified	2	1
Total	39	28

If a condition had both composite and non-composite responder endpoints identified, they were only included in the composite column

^a^Excludes solid tumour oncology (as the utility of the method had previously been highlighted there)

The clinical classifications for which the method appears most useful in terms of number of endpoints are rheumatology (11 found), non-solid tumour oncology (10 found), neurology (9 found) and cardiovascular (8 found).

We note that this review was not systematic and it represents a likely substantial underestimate of the number of clinical areas where suitable endpoints are used, as our review only covered clinical areas which were covered by a COS published by 2016. As an example, Table 1 mentions type II diabetes and shows diabetes remission would be a suitable endpoint; however, since there was no associated COS published by 2016, it does not appear in the identified clinical areas (although gestational diabetes does).

## Discussion

In this paper we have highlighted and summarised previous statistical work on an efficient analysis approach called the augmented binary method, which can be used to improve analysis of composite responder outcomes. The method allows retention of clinically relevant endpoints whilst improving the power of analyses by an amount equivalent to a considerable increase in sample size. As well as describing previous work, we have conducted a review of new clinical areas for which the method could be used. We have also provided a worked example of fitting the model using publicly available R code in the supplementary material.

Through our review of core outcome sets, we have found numerous new disease areas where the augmented binary method could be applied to gain power. We acknowledge that many of the core outcome sets were developed before best practice guidance [20] existed, and therefore their quality may differ. We also do not know whether the augmented binary method is currently being used in any areas; however, our intuition is that it is not—due to the relatively low number of citations of the methodological papers describing the method. We hope that this paper may help improve the uptake of the method.

Although the results indicate the widespread utility of the method, there are several areas where further methodological research is required to fully realise the possible benefits.

There are several endpoints which are typically analysed using time-to-event methods. Many progression, remission and relapse endpoints are used, and the time until such a negative event occurs is the quantity of interest. Although the augmented binary method is well developed for composite responder outcomes that are analysed at a single timepoint or longitudinally, further work is needed to apply it to time-to-event outcomes.

In some cases, the composite responder outcomes are particularly complex, with more than two components and with response being defined as meeting some, but not all, of the criteria. Recent work in this area [16] shows that the potential efficiency gain is even larger in this case. In addition, the method, with some modification to the underlying latent variable model, could be applied in the case of a categorised responder endpoint with more than two levels.

We have focused on how the method can improve the statistical power of trials. An alternative way to use this improved power would be to reduce the sample size needed for a target power level. A barrier to widespread use of the method in this way is sample size estimation. Methods for conducting sample size estimation for trials using responder endpoints analysed using latent variable models are developed in McMenamin et al. [21]. Barriers to this include the requirement for pilot data to inform the values used for the required parameters. Further work on ensuring that these methods can be used in practice, such as software that can be used in a wide variety of situations, is a priority for future research.

## Conclusions

In this paper we have shown that responder composite outcomes are used as primary clinical trial endpoints in many diseases. Analysing data from these trials using the augmented binary approach would improve power equivalent to increasing the sample size by at least 35%. Further methods research is needed to improve time-to-event analyses using these outcomes as events.

## Supplementary information

**Additional file 1.** Supplementary material for ’Analysis of responder-based endpoints: improving power through utilising continuous components’.

## Data Availability

The datasets used are available from the authors of [19] upon request. R code for using the augmented binary method is provided in the supplementary material.

## Abbreviations

CI: Confidence interval
COMET: Core Outcome Measures in Effectiveness Trials
COS: Core outcome set
RCT: Randomised controlled trial
SLE: Systemic lupus erythematosus
