# Supplementary material for: Analysis of responder-based endpoints: improving power through utilising continuous components
Source: Trials. 2020 May 25;21:427. doi: 10.1186/s13063-020-04353-8 (PMC7249409; doi:10.1186/s13063-020-04353-8)
Supplement: Supplementary file 1 — Additional file 1. Supplementary material for ’Analysis of responder-based endpoints: improving power through utilising continuous components’. [file 13063_2020_4353_MOESM1_ESM.docx]

**Supplementary material for “Analysis of responder-based endpoints: improving power through utilising continuous components”**

1. **Supplementary results - details of identified conditions with suitable endpoints for augmented binary use**

The following tables show the newly identified clinical conditions (excluding solid tumour oncology, rheumatoid arthritis, and systemic lupus erythematosus (which had previously been identified as areas where the method could be used). In each case, if not otherwise stated, response requires all of the conditions to be met.

Table 1a – list of bleeding and transfusion conditions where suitable endpoints are used; *denotes a single dichotomized continuous variable

| **Condition** | **Endpoint** | **Response definition** |
| --- | --- | --- |
| Anemia | Recurrence of infarct or hemorrhage | One of:   - New silent cerebral infarct - Existing silent cerebral infarct increased in size by 3mm in any linear dimension - hemorrhage |
| Immune thrombocytopenic purpura | Complete response | - Platelet count between 30 and 100 × ${10}^{9}$/L - at least doubling of the baseline count |
| Transfusion | Failure* | - 1 hour Corrected Count Increment<7.5 |

Table 1b – list of cancer (excluding solid-tumour oncology) conditions where suitable endpoints are used; *denotes a single dichotomized continuous variable

| **Condition** | **Endpoint** | **Response definition** |
| --- | --- | --- |
| Acute Myeloid Leukaemia | Response | Meet all of:   - < 5% blasts in the bone marrow - No blasts with Auer rods - Normal maturation of all cellular components in the bone marrow - No extramedullary disease (e.g., CNS, soft tissue disease) - Neutrophils ≥ 1,000/µL - Platelets ≥ 100,000/µL - Transfusion independent |
| Breast cancer related lymphedema | Excess arm volume* | - ≥ 50% reduction |
| Dyspnea or Breathlessness in Palliative Care | Severe breathlessness* | - Numerical Rating Scale mild or moderate (NRS ≤ 6) |
| Fever and neutropenia from cancer treatment | Fever* | - Body temperature >37.5 degrees centigrade |
| Hodgkin’s disease and lymphoma | Complete response | - complete normalization of FDG-PET uptake (Deauville score of 1 to 3)   or   - complete resolution of all target lesions for non-FDG avid lymphoma |
| Malignant lymphoma | Progression-free-survival (PFS) | - Duration from start of the treatment to: - Target tumour lesions longest diameter increasing 20% from nadir; - New lesions - Death. |
| Myeloma (Newly diagnosed) | PFS and Very Good Partial Response (VGPR) | - Serum and urine M-component detectable by immunofixation but not on electrophoresis   or   - ≥ 90% reduction in serum M-component plus urine M-component <100mg/24h |
| Myeloma (Refractory) | Complete response | - No M protein on serum and urine immunofixation - Disappearance of any soft-tissue plasmacytomas - < 5% plasma cells in bone marrow |
| Myeloma (Relapsed) | PFS | Defined as in malignant lymphoma |
| Systematic light-chain amyloidosis | Haematological response* | Reduction in dFLC (difference between involved and uninvolved circulating free light chains) >50% from baseline. |

Table 1c – list of cardiovascular/circulation conditions where suitable endpoints are used; *denotes a single dichotomized continuous variable

| **Condition** | **Endpoint** | **Response definition** |
| --- | --- | --- |
| Aortic dissection | Procedural success | All of:   - Successful introduction and deployment of device; - absence of the need for conversion to open surgery or immediate endovascular reintervention; - absence of aortic death, graft thrombosis, obstruction, twisting or kinking - absence of graft migration>10 mm proximally ordistally, - absence of failure of graft integrity, absence of aneurysm rupture - absence of increase in aortic diameter>5 cm within 30 days of the baseline procedure |
| Aortic valve implantation | Clinical efficacy | - All-cause mortality - All stroke (disabling and non-disabling) - Requiring hospitalizations for valve-related symptoms or worsening congestive heart failure - NYHA class III or IV - Valve-related dysfunction (mean aortic valve gradient ≥20 mmHg, EOA ≤0.9– 1.1 cm and/or DVI <0. 35 m/s, AND/OR moderate or severe prosthetic valve regurgitationa) |
| Aortic valve stenosis | Device success | - Absence of procedural mortality - Correct positioning of a single prosthetic heart valve into the proper anatomical location - Intended performance of the prosthetic heart valve (no prosthesis– patient mismatcha and mean aortic valve gradient <20 mmHg or peak velocity <3 m/s) - no moderate or severe prosthetic valve regurgitationa |
| Atrial fibrillation | Atrial fibrillaton control* | - ≥ 90% reduction in AF burden |
| Chronic leg admea | Normal range of motion* | - E.g. Knee flexion (0-130°), Knee extension (120-0°) |
| Deep venous thrombosis and pulmonary embolism | Major bleeding | - No fatal bleeding - No clinically overt bleeding associated with a decrease in Hgb of at least 20${gL}^{-1}$ - No bleeding that is retroperitoneal, pulmonary, intracranial, or otherwise involves the central nervous system - No bleeding that requires surgical intervention in an operating suite. |
| Head and neck lymphatic malformation | Response* | - ≥ 80% regression in vascular malformation |
| Mitrial regurgitation | Severe mitrial regurgitation | All of:   - >=40mm^2^ effective regurgitant orifice area - >=60mm^2^ regurgitant volume - Dilated Left ventricular and left atrium size - Pulmonary artery systolic pressure >50 at rest without other cause. |

Table 1d – list of dentistry and vision conditions where suitable endpoints are used; *denotes a single dichotomized continuous variable

| **Condition** | **Endpoint** | **Response definition** |
| --- | --- | --- |
| Intermittent exotropia | Alignment, Deterioration | - <10 esoporia/tropia   or   - <10 exophoria/tropia |
| Missing teeth (Edentulous) | Implant success | All of:   - probing pocket depth <5 mm - absence of bleeding/suppuration on probing - no additional bone loss. |
| Peridontal disease | Mobility* | - Grade 1 (<1mm horizontal movement) |

Table 1e – list of gastroenterology conditions where suitable endpoints are used; *denotes a single dichotomized continuous variable

| **Condition** | **Endpoint** | **Response definition** |
| --- | --- | --- |
| Crohn’s Disease | Clinical remission | - Crohn’s Disease Activity Index below a threshold (e.g. 150) - No use of steroids or rescue treatment |
| Nonalcoholic steatohepatitis | Resolution of Steatohepatitis without fibrosis | - Improvement in NAS of two points - No worsening of fibrosis |
| Reflux chest pain syndrome trial | Chest pain* | - Reduction of ≥ 50% in symptom score |
| Reflux oesophagitis syndrome trial | Troublesome regurgitation | Both of:   - Severity score >2 - Frequency score >3   On the Reflux Disease Questionnaire |

Table 1f – list of infectious disease conditions where suitable endpoints are used

| **Condition** | **Endpoint** | **Response definition** |
| --- | --- | --- |
| Influenza | Resolution of fever | Both of:   - Temperature ≤37.8°C - No subjective symptoms of fever for 24 hours |
| Intraabdominal infection | Recovery | At least four of:   - Acute physiology score ≤ 2 - Temperature ≤ 37.8°C for 24h - Gastrointestinal motor activity has returned - Gastrointestinal function is present - Mental status has returned to pre-illness baseline |
| Pneumonia | Clinical stability | - Temperature ≤37.8°C - Heart rate ≤100 beats/min - Respiratory rate ≤24 breaths/min - Systolic blood pressure ≥90mmHg - Arterial oxygen saturation ≥90% or a partial pressure of oxygen ≥60mmHg on room air - Normal mental status |

Table 1g – list of lungs and airways conditions where suitable endpoints are used; *denotes a single dichotomized continuous variable

| **Condition** | **Endpoint** | **Response definition** |
| --- | --- | --- |
| Connective tissue disease associated interstitial lung disease | Decline in forced vital capacity* | - Reduction in FVC >0 |
| Idiopathic pulmonary fibrosis | Decline in forced vital capacity* | - Reduction in FVC >0 |

Table 1h – list of mental health and addiction disorders where suitable endpoints are used; *denotes a single dichotomized continuous variable

| **Condition** | **Endpoint** | **Response definition** |
| --- | --- | --- |
| Alcohol abuse | Proportion of heavy drinking days | Heavy drinking day:   - 4/5 drinks for women - 5/6 drinks for men |
| Bipolar disorder (children and adolescents) | Response* | - Children’s depression rating scale reduction of >=50% |
| Major depressive disorder | Response | - Clinician's Global Improvement ≤2 - Hamilton Rating Scale for Depression ≤10 |
| Nicotine abuse | Abstinence (objectively confirmed) | - Self-report of no smoking during the previous 7 days - Saliva cotinine level of <20 ng/ml |

Table 1i – list of neurology disorders where suitable endpoints are used; *denotes a single dichotomized continuous variable

| **Condition** | **Endpoint** | **Response definition** |
| --- | --- | --- |
| Cerebral Palsy | Modified Teacher's Drooling Scale* | - ≥3 point change |
| Chronic Inflammatory Demyelinating Polyradiculoneuropath | Impairment* | - Improvement ≥1 in Inflammatory Neuropathy Cause and Treatment disability score |
| Headache | Primary headache dependent measure e.g. HA index* | - >50% improvement |
| Hypoxic-ischemic brain injury | Moderate-severe disability | - Bayley MDI between 1-2SD below mean   and   - one or more of the following: Level 2 on GMF, deafness with no amplification |
| Intracranial cerebral atherosclerosis | Acute & subacute arterial occlusions with or without ischemic stroke | E.g.   - < 2/3 of entire vascular territory is visualised - Increase ≤4 points on NIHSS |
| Multifocal Motor Neuropathy | Impairment* | - MCID-SE score ≥1.96 |
| Multiple Sclerosis | Progression of disability* | - Worsening in EDSS score from baseline to 12 weeks. |
| Pain | Proportion of individuals with improvement in pain* | - >30% reduction in numeric pain rating scale |
| Traumatic brain injury | Severe disability rating* | - None (0), Mild (1), Partial (2-3), Moderate (4-6), Moderately severe (7-11), Severe (12-16), Extremely severe (17-21), Vegetative state (22-24), Extreme vegetative state (25-29) |

Table 1j – list of orthopaedics and trauma conditions where suitable endpoints are used; *denotes a single dichotomized continuous variable

| **Condition** | **Endpoint** | **Response definition** |
| --- | --- | --- |
| ACL injury | Knee function* | - >10 point change in Knee injury and Osteoarthritis Outcome Score (KOOS) |
| Burns | Response* | - > 0.5 change in 10 point Kapandji thumb scale |
| Dupuytren's disease | Contracture recurrence | - Increase in joint contracture of ≥30° in presence of palpable cord   or   - Patient underwent repeat intervention to correct new/worsening contracture in the treated joint |
| Low back pain | Severe disability* | - Oswestry disability score:   0-20% minimal,  21-40% moderate,  41-60% severe,  61-80% crippled,  81-100% bed-bound |

Table 1k – list of renal and urological conditions where suitable endpoints are used; *denotes a single dichotomized continuous variable

| **Condition** | **Endpoint** | **Response definition** |
| --- | --- | --- |
| Acute kidney injury | Proportion with acute kidney injury | - rise in serum creatinine level of at least 2-fold;   or   - a serum creatinine level of >3.96 mg/dL with an increase of >0.5 mg/dL. |
| Acute renal failure | Proportion with acute renal failure | ACF is defined as:   - Increase SCreat x3 - 75% decrease in glomerular filtration rate   or   - SCreat ≥4mg/dl |
| Male sexual dysfunction | Severe dysfunction* | - <14 on International Index of Erectile Function |

Table 1l – list of rheumatology conditions (excluding rheumatoid arthritis and systemic lupus erythematosus) where suitable endpoints are used; *denotes a single dichotomized continuous variable

| **Condition** | **Endpoint** | **Response definition** |
| --- | --- | --- |
| Acute Gout | Proportion of patients who responded* | - sUA level of <6.0mg |
| Ankylosing spondylitis | ASAS20 response | - 20% improvement and ≥ 10 units of change (on a 0–100 scale) in each of 3 domains - No worsening of a similar amount in the fourth domain   (Components are physical function, pain, inflammation and patient’s global assessment) |
| Idiopathic arthritis-associated uveitis | Best corrected visual acuity above threshold and no light perception | - Best-corrected visual acuity, thresholds ≤20/50, ≤20/200 - no light perception - Estimate contribution of amblyopia, yes/no |
| Juvenile arthritis | Response | Improvement by 30% in at least 3 of:   - MD global assessment; - parent or patient global assessment - functional ability; - number of joints with active arthritis; - number of joints with limited range of motion; - Erthrocyte sedimentation rate) |
| Juvenile dermatomyositis | Responder index | - ≥ 4 point reduction from baseline in safety of estrogen in lupus national assessment (SELENA) systemic lupus erythematosus disease activity index (SLEDAI) score - No worsening (increase of <0.30 points from baseline) in physician's global assessment (PGA) - No new British Isles Lupus Assessment Group of SLE clinics (BILAG) A organ domain score or 2 new BILAG B organ domain scores compared with baseline |
| Prevention of fracture in high risk populations | Response | - Bone mineral density increase - Occurrence of new vertebral fractures |
| Proliferative and membranous lupus renal disease | Urinary protein levels within normal range* | - Between 6 and 8.3 grams per deciliter (g/dL) |
| Sarcopenia prevention | Occurrence of sarcopenia | Heterogeneity in precise definition, but severe sarcopenia defined by all of the following:   - Low muscle strength (assessed with chair stand test or grip strength) - Low muscle quantity/quality - Low physical performance as assessed with gait speed test or short physical performance battery |
| Sjogren's syndrome | Response | - >30% reduction in analog scales evaluating dryness, pain and fatigue |
| Systemic Sclerosis | SCP in normal range, no renal crisis | E.g.   - < 3.0mg/dl not drug related - No renal crisis |
| Vasculitis disorders | Response/partial improvement* | - 50% improvement in disease activity score. |

Table 1m – list of other conditions where suitable endpoints are used; *denotes a single dichotomized continuous variable

| **Condition** | **Endpoint** | **Response definition** |
| --- | --- | --- |
| Endometriosis-related pain | Response | - >30% reduction in symptom score - No use of rescue analgesics |
| Gestational diabetes mellitus | Gestational hypertension | One of:   - Systolic blood pressure >=140 - Diastolic blood pressure >=90 - Taking anti-hypertensive medication |
| Neurofibromatosis | Severe pain* | - Pain is severe if ≥7 on Numerical Rating Scale-11 (11-point scale) |

**2. Technical explanation of fitting the method**

We illustrate the technical details for using the method in the situation where the responder endpoint is formed from a binary component and a continuous component. More advanced situations are described in: Wason and Jenkins^1^ (two follow-up times), Lin and Wason^2^ (more than two follow-up times using a simplified method) McMenamin et al^3^ (more than two components).

This explanation is accompanied by an R package, AugBin, which is available for download from the following address: <https://github.com/mjg211/augbin>. This can be installed in R with the ‘devtools’ library:

>library(devtools)

>install_github("mjg211/augbin")

This package contains a function *augbin* which allows the user to supply information on a continuous component, a binary component, treatment allocation and dichotomization threshold (demonstrated in section 3). It returns the estimated odds ratio between experimental and control treatment, together with 95% confidence interval.

We assume a total of N patients are included in the study, with $i\in\{1,\ldots,N\}$ labelling the patient. The dichotomisation threshold, *d*, is specified to determine what values of the continuous outcome are required to be a responder.

For patient *i*, we define the following notation:

1. $T_{i}\in\{0,1\}$ denoting which treatment arm the patient was randomised to (0 being control and 1 being experimental);
2. $Y_{i}$ denoting the continuous component of the responder outcome;
3. $Z_{i}$ denoting the binary component of the responder outcome;
4. $R_{i}$ denoting whether the patient is a responder or non-responder.

We use the responder definition that $R_{i}=1$ if $Y_{i}<d, Z_{i}=0$, i.e. that the continuous component is below the dichotomisation threshold and the binary component is 0. If requiring the continuous component to be above a threshold, then one can work with -$Y_{i}$ and -*d* to fit in with the above definition. If the binary component should take value 1, then working with 1-$Z_{i}$ for the binary component will fit with the above.

We fit a latent variable model to the data $\{\left( Y_{1},Z_{1} \right),\ldots,\left( Y_{N},Z_{n} \right)\}$. This makes the modelling assumption that the binary component relates to whether or not an underling normally distributed latent variable $Z_{i}^{*}$ is above or below 0, i.e: $Z_{i}=I\{Z_{i}^{*}$ >0}.

The model then is as follows:

where the residual errors are correlated and distributed as follows:

Note that to ensure identifiability, the variance of the latent variable is set to 1.

In order to make inference about the parameters $\theta=(\alpha_{1},\alpha_{2},\beta_{1},\beta_{2},\sigma_{1},\rho)$ we define a likelihood, with contribution from individual *i*:

$$l\left( \theta;Y_{i},Z_{i},T_{i} \right)=f\left( Y_{i},Z_{i}^{*} | T_{i};\theta\right)$$

$=f\left( Y_{i} | T_{i};\theta\right)f(Z_{i}^{*}|Y_{i},T_{i};\theta)$.

This decomposes the joint likelihood as the product of the marginal probability density function (pdf) of the continuous component multiplied by the conditional distribution of the latent binary component given the continuous component.

The marginal pdf of $Y_{i}$ is normal with mean $\alpha_{1}+\beta_{1}T_{i}$ and variance $\sigma_{1}^{2}$. By standard properties of the multivariate normal distribution, the conditional distribution $Z_{i}^{*}|Y_{i}$ is normal with mean $\tilde{\mu}$ and variance  $\tilde{\sigma}_{2}^{2}$, where:

$$\tilde{\mu}=\alpha_{2}+\beta_{2}T_{i}+\frac{\rho}{\sigma_{1}}\left( Y_{i}-\alpha_{1}-\beta_{1}T_{i} \right)$$

$$\tilde{\sigma}_{2}^{2}=1-\rho^{2}$$

Since $Z_{i}^{*}$ is not observed directly, the contribution of patient i to the likelihood is:

$$f\left( Y_{i} | T_{i};\theta\right)\int_{A\left( Z_{i} \right)} g\left( x,\tilde{\mu},\tilde{\sigma}_{2}^{2} \right)dx,$$

where $A\left( Z_{i} \right)=\left( 0,\infty\right)$ if $Z_{i}=1$ and $\left( -\infty,0 \right)$ if $Z_{i}=0$, and g is the pdf of a normal distribution with mean $\tilde{\mu}$ and variance $\tilde{\sigma}_{2}^{2}$.

The likelihood for the entire set of data can then be written as the product of individual contributions from each patient and maximised over $\theta$ to find the maximum likelihood estimator, $\hat{\theta}$. The overall optimisation in augbin is done using the BFGS option in the R function optim.

In practice to make the optimisation easier, we use transformed parameters for the variance parameters $\sigma_{1}^{2}$ and $\rho$: $\sigma_{1}^{2}=\exp\left( \delta_{1} \right),\rho=2\frac{exp(\delta_{2})}{1+exp(\delta_{2})}-1$.

These reparameterisations mean that we are maximising over parameters that can take any real value but enforcing the requirements for the variance and correlation parameters.

Once the maximum likelihood estimator, $\hat{\theta}$, and the covariance matrix Cov($\hat{\theta}$) are found, we can find the probability of response for participants who are given the control and experimental treatment:

$$p\left( t_{i};\theta\right)=\int_{-\infty}^{0} \int_{-\infty}^{d} g_{2}\left( \left( x_{1},x_{2} \right),\bar{\mu}(t_{i}),\bar{\Sigma} \right)dx_{2}dx_{1},$$

where $\bar{\mu}=\left( \begin{matrix} \alpha_{1}+\beta_{1}t_{1} \\ \alpha_{2}+\beta_{2}t_{1} \end{matrix} \right)$ and $\bar{\Sigma}= \left( \begin{matrix} \sigma_{1}^{2} & \rho\sigma_{1} \\ \rho\sigma_{1} & 1 \end{matrix} \right)$

Since we are typically interested in the difference in effect between two arms, we can work with some measure of the difference between $p_{1}= p\left( t_{i}=1;\theta\right)$ and $p_{0}=p\left( t_{i}=0;\theta\right)$. There are various options for this, but all work in a similar way. Here (and in the R code), we use the odds ratio. Due to the log odds-ratio being symmetric around 0, it is more convenient to work with that and then transform back to the odds-ratio scale. The log odds-ratio is:

$$\mathrm{LOR}\left( \theta\right)=\log\left( \frac{p_{1}\left( \theta\right)}{1-p_{1}\left( \theta\right)} \right)-\log\left( \frac{p_{0}\left( \theta\right)}{1-p_{0}\left( \theta\right)} \right).$$

We can estimate the log odds ratio by $\mathrm{LOR}\left( \hat{\theta} \right)$ and its variance by applying the delta method:

$$Var\left( \mathrm{LOR}\left( \hat{\theta} \right) \right)\approx\left( \nabla LOR\left( \hat{\theta} \right) \right)^{T}Cov\left( \hat{\theta} \right)\left( \nabla LOR\left( \hat{\theta} \right) \right),$$

where $\nabla LOR\left( \hat{\theta} \right)$ is the vector of partial derivatives of $LOR(\theta)$ evaluated at $\hat{\theta}$.

This process can be used to form a 95% confidence interval for the log odds-ratio:

$$\left( \mathrm{LOR}\left( \hat{\theta} \right)-1.96\sqrt{Var\left( \mathrm{LOR}\left( \hat{\theta} \right) \right)},\mathrm{LOR}\left( \hat{\theta} \right)+1.96\sqrt{Var\left( \mathrm{LOR}\left( \hat{\theta} \right) \right)} \right).$$

If there is missing data in one or other component, then a complete cases analysis (making a missing completely at random assumption) or a multiple imputation (making a missing at random assumption) could be applied. In the latter case, the augmented binary method could be applied to each imputed dataset and then results combined using Rubin’s rules.

Covariates can also be included in the model as described in Wason and Seaman^4^. This changes $p_{1}\left( \theta\right)$ and $p_{0}\left( \theta\right)$ so that they are the mean fitted probabilities from the model across all patients (allowing for covariate dependence).

**3. Demonstration of augbin code on simulated data**

This approach is implemented in the augbin function. As an example, the following code simulates a dataset (set.seed is used so it is reproducible). Note that this was ran on R v3.6.1 on Mac OS Catalina on 11^th^ December 2019. It is possible that future versions of augbin may change syntax.

> install_github("mjg211/augbin")

>library(augbin)

> set.seed(1)

> #simulate continuous component (Y),binary component (Z) and treatment indicator (T)

> Y=rnorm(100)

> Z=rbinom(100,1,0.4)

> T=rbinom(100,1,0.5)

> #dichotomisation threshold set to 0

> dichotomisationthreshold=0

> #class patients as responders or not

> responder=ifelse(Y<0 & Z==0,1,0)

> #get odds ratio and confidence interval from binary method:

>

> glm.binary=glm(responder~T,family="binomial")

> dataforaugbin= build_augbin_data(continuous=Y,binary=Z,treatment=T,dichotomisation = 0)

>CI.binary=as.double(exp(confint(glm.binary)[2,]))

Waiting for profiling to be done...

> augbin.out=augbin(dataforaugbin

> print(CI.binary)

[1] 0.4111575 2.2378300

> print(as.double(augbin.out$odds_ratio[2:3]))

[1] 0.4878662 1.7288904

Note that for this simulated dataset the width from the augmented binary method is narrower than from the traditional method treating the responder outcome as binary.

Exploring the average width can be done with the following code:

set.seed(1)

CI.binary=matrix(0,1000,2)

CI.binary.highersamplesize=matrix(0,1000,2)

CI.augbin=matrix(0,1000,2)

for(iteration in 1:1000)

{

#simulate continuous component (Y),binary component (Z) and treatment indicator (T)

Y=rnorm(100)

Z=rbinom(100,1,0.4)

T=rbinom(100,1,0.5)

#class patients as responders or not

responder=ifelse(Y<0 & Z==0,1,0)

#get odds ratio and confidence interval from binary method:

glm.binary=glm(responder~T,family="binomial")

CI.binary[iteration,]=as.double(exp(confint(glm.binary)[2,]))

CI.augbin[iteration,]=as.double(augbin(build_augbin_data(continuous=Y,binary=Z,treatment=T,dichotomisation = 0))$odds_ratio[2:3])

}

#repeat binary method for larger sample size

for(iteration in 1:1000)

{

#simulate continuous component (Y),binary component (Z) and treatment indicator (T)

Y=rnorm(135)

Z=rbinom(135,1,0.4)

T=rbinom(135,1,0.5)

#class patients as responders or not

responder=ifelse(Y<0 & Z==0,1,0)

#get odds ratio and confidence interval from binary method:

glm.binary=glm(responder~T,family="binomial")

CI.binary.highersamplesize[iteration,]=as.double(exp(confint(glm.binary)[2,]))

}

print(mean(CI.binary[,2]-CI.binary[,1]))

[1] 2.267385

print(mean(CI.augbin[,2]-CI.augbin[,1]))

[1] 1.482062

print(mean(CI.binary.highersamplesize[,2]-CI.binary.highersamplesize[,1]))

[1] 1.766267

Note that the average width of the CI from the augmented binary method is narrower even than if the traditional method was used with a 35% larger sample size.

**References**

1. Wason JMS, Jenkins M. Improving the power of clinical trials of rheumatoid arthritis by using data on continuous scales when analysing response rates: an application of the augmented binary method. *Rheumatology*. 2016;55(10):1796-1802.

2. Lin C-J, Wason JMS. Improving phase II oncology trials using best observed RECIST response as an endpoint by modelling continuous tumour measurements. *Stat Med*. 2017;36(29):4616-4626. doi:10.1002/sim.7453

3. McMenamin M, Barrett JK, Berglind A, Wason JMS. Employing latent variable models to improve efficiency in composite endpoint analysis. February 2019.

4. Wason JM, Seaman SR. A latent variable model for improving inference in trials assessing the effect of dose on toxicity and composite efficacy endpoints. *Stat Methods Med Res*. February 2019:096228021983103. doi:10.1177/0962280219831038
